# Reducing effect of insulin resistance on alpha-synuclein gene expression in skeletal muscle

**DOI:** 10.1186/s13098-019-0499-6

**Published:** 2019-12-02

**Authors:** Amirhosein Khoshi, Golnaz Goodarzi, Rezvan Mohammadi, Roghaye Arezumand, Meysam Moghbeli, Mahnaz Najariyan

**Affiliations:** 1 Department of Clinical Biochemistry, School of Medicine, North Khorasan University of Medical Sciences, Arkan Roadway, Bojnurd, Iran; 2 Department of Biochemistry, School of Medicine, Tehran University of Medical Sciences, Tehran, Iran; 3 Department of Biotechnology, School of Medicine, North Khorasan University of Medical Sciences, Bojnurd, Iran; 4 Department of Biotechnology, School of Medicine, Shaheed Beheshti University of Medical Sciences, Tehran, Iran; 5 Medical Genetics Research Center, Mashhad University of Medical Sciences, Mashhad, Iran; 6 Student Research Committee, School of Medicine, North Khorasan University of Medical Sciences, Bojnurd, Iran

**Keywords:** Insulin resistance, Alpha-synuclein, Muscle tissue, C2C12 muscle cells, Type 2 diabetes

## Abstract

**Background:**

Alpha-synuclein (SNCA) as the presynaptic protein is expressed in different tissues and prevents insulin-resistance (IR) through increasing glucose-uptake by adipocytes and muscles. However, the effect of insulin metabolism on SNCA expression has scarcely elucidated. In present study we assessed the probable effect of insulin resistance on SNCA expression in muscle C2C12 cells and also skeletal muscle tissues of type 2 diabetic mice.

**Materials and methods:**

Sixteen male C57BL/6 mice were divided into two experimental groups, including control and type 2 diabetic mice with IR (induced by high-fat diet + low-dose streptozotocin). The animals of the study involved the measurements of fasting blood glucose, oral-glucose-tolerance-test, as well as fasting plasma insulin. Moreover, insulin-resistant and insulin-sensitive muscle C2C12 cells were prepared. The insulin-resistance was confirmed by the glucose-uptake assay. Comparative quantitative real time PCR was used to assess the *SNCA* expression.

**Results:**

The obtained results have showed a significant ~ 27% decrease in *SNCA* expression level in muscle tissue of diabetic mice (P = 0.022). Moreover, there was a significant change of *SNCA* expression in insulin-resistant C2C12 cells (P < 0.001).

**Conclusion:**

Type 2 diabetes due to insulin-resistance can decrease *SNCA* gene expression in muscles. In addition to the role of SNCA in cell susceptibility to insulin and glucose uptake, the SNCA expression can also be affected by insulin metabolism.

## Background

Insulin has a critical role in regulation of glucose uptake and metabolism in different tissues via activating of the tyrosine kinase signaling pathway [1]. Insulin resistance (IR) as the main hyperglycemic process in non-insulin dependent diabetes mellitus (NIDDM) is observed in many tissues such as adipose and skeletal muscle [2]. Alpha-synuclein (SNCA, α-Syn) as one of the members of synuclein protein family is mainly expressed in brain tissue. Synucleins have 127–140 amino acids with similar domains and sequence similarity of 62–55% [3, 4]. SNCA as a principal component of Lewy bodies can be associated with neurodegenerative disorders such as Parkinson’s disease [5, 6]. However, it has been shown that the SNCA is expressed not only in neurons but also in various tissues including liver, spleen, kidney, red blood cells, cardiomyocytes, and skeletal muscles [7]. SNCA increases the glucose uptake in adipocytes and skeletal myocytes via activation of LPAR2/Gab1/PI3K/Akt signaling pathway and can be involved in prevention of insulin resistance [1, 3, 8]. Moreover, SNCA increases the transfer of vesicles containing GLUT4 to the dopaminergic neurons cell surface through its interaction with Rab8 [9]. It has reported that decreased levels of plasma alpha-synuclein were associated with insulin resistance. Furthermore, the plasma level of alpha-synuclein was reversely related to body mass index (BMI) and homeostatic model assessment for insulin resistance (HOMA-IR) [1]. However, there is not any clear principal mechanism of SNCA interactions with molecules in the insulin-dependent intracellular pathway. Since the basal expression of *SNCA* in the muscle cells in response to insulin has not been investigated to date, we decided to examine the expression of *SNCA* in insulin-resistant (IR) and insulin-sensitive (IS) models of skeletal muscle cells in both in vitro and in vivo studies.

## Materials and methods

### Animals study

This study was conducted on a total of 16 male C57BL/6 mice (Pasteur Institute, Iran) with 8 weeks of age and approximately 20–25 g. All procedures were according to the institutional animal ethics guidelines which approved by the Ethics Committee of North Khorasan University of Medical Sciences (ethical code: IR.nkums.REC.1397.036). The animals were kept in a clean cage under controlled condition (25 ± 2 °C) and humidity (50%) with a 12/12 h light/dark cycle. All the mice were fed with a normal pellet diet (NPD) containing 5% fat, 50% carbohydrate, 25% protein and total calorific value 25 kJ/kg (Royan Institute, Iran) and free water 1 week before the initiation of the experiment and allowed to acclimatize to the laboratory environment. All the mice were divided into two groups with eight animals for each experimental groups as given below: group (1) healthy mice as controls fed with normal chow, group (2) the mice with diebetes induced by high-fat diet + STZ (HFD + STZ).

### Type 2 diabetes induction

After 7 weeks of dietary manipulation of diabetic group by high-fat diet, a low dose of STZ (45 mg/kg) was injected into the mice, intraperitoneally. The food intake, body weight, and fasting plasma glucose were measured every week until 12 weeks. The mice with a fasting blood glucose level higher than 8.3 mmol/L 4 weeks after the injection were included to the study [10].

### Biochemical measurements

As we mentioned in previous study, an oral glucose tolerance test was performed following 14-h fasting in the study groups 4 weeks after STZ injection. The glucose concentrations were measured in blood samples that were taken from the tail using Accu-Chek glucose meter at 0, 15, 30, 60 and 120 min post administration of glucose solution (3 g/kg) [11]. Moreover, the concentrations of fasting plasma insulin (Abnova, Taiwan) were measured by the enzyme-linked immunosorbent assay (ELISA) following the manufacturer’s protocols at the end of the animal study [12, 13].

### Cell culture and differentiation

The C2C12 cells (Pasteur Institute, Iran) were cultured in a growth medium (DMEM, Gibco, UK) supplemented with 10% heat-inactivated fetal bovine serum (FBS, Gibco, UK), penicillin 100 IU/ml, and streptomycin 100 μg/ml (Gibco, UK) before cell differentiation (37 °C and 5% CO_2_). After seeding into 12-well plates and obtaining 60% of confluency, myoblasts differentiation to myotubes was induced by changing growth medium (GM) to differentiation medium (DM) which was supplemented with 3% of horse serum (Capricorn Scientific, Germany) and 1% of penicillin/streptomycin. Insulin-resistant (IR) cells were generated through a cell culture for 3 days in differentiation medium supplemented with 100 nM insulin (Sigma, USA). The insulin-sensitive (IS) cells were not exposed to insulin.

### Glucose uptake assay

Glucose uptake was assessed in IR and IS models to confirm the insulin-resistance. Myoblasts were initially incubated for 1 h in glucose-free media followed by 3 h incubation in DMEM containing 8 mM glucose. Then the cells were exposed to 1 μM insulin for 1 h and media were taken from the respective wells. The remained glucose in media was measured using the glucose oxidase method (Pars Azmoon, Iran) [14].

### RNA extraction and quantitative real-time PCR

RNA extractions from cells and frozen muscle tissues were performed using a column method according to the manufacturer’s instruction (Bio basic, Canada). The total RNA quality and concentrations were determined by measuring the 260/280 nm ratio using a bio-spectrophotometer (Lambda max, Japan). Complementary DNA (cDNA) was synthesized using PrimeScript Reverse Transcript Reagent Kit (Takara, Japan). The quantitative SYBR green (Takara, Japan) real-time polymerase chain reaction (qRT-PCR) was done in duplicate reactions (Rotor-Gene 6000, Qiagen, Germany) to assess the *SNCA* mRNA expression. All the primer sequences are mentioned in Table 1. Thermal profile was a 95 °C for 5 min followed by 40 cycles at 95 °C for 10 s, 60 °C for 20 s, and 62 °C for 30 s. Melting curve analysis was also performed by increasing the temperature (1 °C) from 57 °C to 95 °C with continues fluorescence acquisition. Relative expressions of *SNCA* mRNA levels were calculated using the 2^−ΔΔCT^ method and normalized based on *Beta 2 Microglobulin* (*B2M)* mRNA levels.

**Table 1:** Specific primers for real time- PCR assay

Primer sequence (5′ → 3′)	Target	PCR product (bp)
F: TGACAGCAGTCGCTCAGAAG R: TCATAGTCTTGGTAGCCTTCCTCTG	*SNCA* NM_001042451	190
F-5′-CTTCAGCAAGGACTGGTC-3′ R-5′-TCTCGATCCCAGTAGACG-3′	*B2M* NM_009735.3	129

### Statistical analysis

All of the in vitro experiments have been repeated three times in duplicate reactions (n = 6). Data were interpreted based on mean ΔC_T_ ± SE. Relative gene expression data comparisons were performed using t-student and ANOVA compared with the control group. Probability values < 0.05 were considered statistically significant.

## Results

### Biochemical parameters

**Fig. 1:**
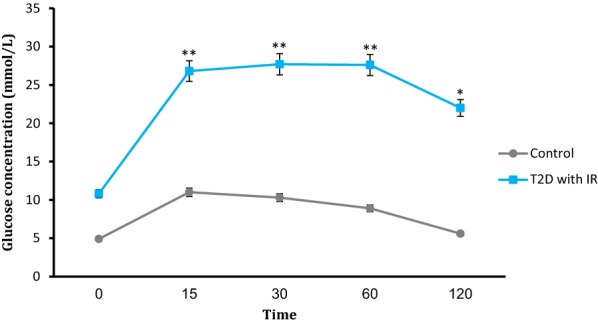
Variations of glucose concentrations after oral glucose tolerance test in insulin resistant diabetic mice compared with healthy control

Feeding the mice with high-fat diet showed significantly increases in FBG and body weight in diabetic group. Injection of STZ at 7th week induced hyperglycemia and decreased body weight in diabetic group. However, the total body weight of HFD + STZ diabetic mice was more than NPD healthy mice. Moreover, the amounts of water and food consumption of diabetic mice were increased compared with control group. Fasting plasma glucose concentrations and fasting plasma insulin levels in diabetic mice were more than healthy group, which together show impaired glucose tolerance and insulin resistance. Moreover, the blood glucose levels after an oral glucose tolerance test in the study groups have shown the rate of glucose disappearance in NPD-fed healthy group was significantly higher than HFD-fed diabetic mice (Fig. 1).

### SNCA gene expression in skeletal muscle tissues and muscle C2C12 cells

**Fig. 2:**
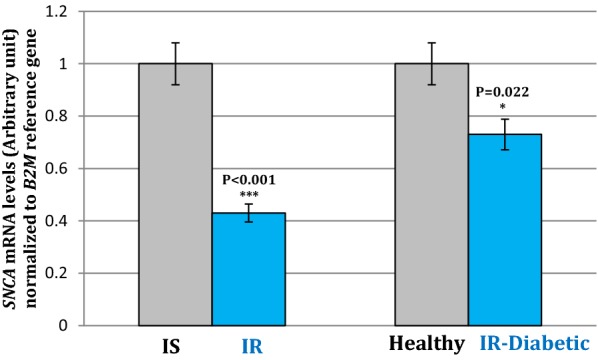
Comparison of the SNCA mRNA expression levels in the insulin resistant (blue) and insulin sensitive states (gray). Left chart shows the changes in SNCA expression in insulin resistant (IR) C2C12 cells compared with insulin sensitive (IS) cells, while right chart shows the changes in SNCA expression in skeletal muscle tissue of insulin resistant diabetic mice compared with healthy control group

We examined the changes on the gene expression of *SNCA* in insulin resistant diabetic mice. As results showed the *SNCA* gene expression decreased ~ 27% in diabetic mice which were induced by HFD with low doses of STZ (P = 0.022) (Fig. 2). Moreover, it was observed that there was a significant ~ 47% decrease in the levels of *SNCA* mRNA expression in IR cells (P < 0.001) compared with IS cells (Fig. 2).

## Discussion

There are little studies that have shown the interactive effects between insulin metabolism and alpha-synuclein (SNCA). This study elucidated a different expression of SNCA in insulin-resistant model of skeletal muscle using the C2C12 cell line and animal study.

Insulin resistance as one of the main T2D features can be developed by various environmental factors such as obesity [15]. Insulin increases the glucose uptake through increasing the glucose transporter 4 (GLUT4) transfer at the cell surface in adipose and skeletal muscle cells. As same as adipose tissues, GLUT4 has a key role in glucose uptake in the muscle fibers [16].

SNCA is mainly expressed in the brain and can be associated with pathogenesis of neurodegenerative disorders such as Alzheimer’s disease, Parkinson’s disease, dementia with Lewy bodies, and multiple-system atrophy [5, 6, 17, 18]. Recent studies have suggested that the SNCA is also associated with physical and pathological processes of muscle tissues [19]. SNCA is involved in regulation of glucose uptake in adipocytes and muscle cells through activation of LPAR2/Gab1/PI3K/Akt signaling pathway. In contrast, lack of SNCA in mice model resulted in reduced glucose uptake and metabolism. Therefore, SNCA can be associated with glucose transfer into the adipocyte and muscle cells through the PI3K/Akt signaling pathway and insulin independent [3]. It has been reported that the insulin resistance was eliminated in adipose and skeletal muscle tissues of the *SNCA* knock-out mice which were fed with high-fat diet. Moreover, they have shown that the plasma levels of SNCA were diminished in patients with insulin resistance. They also showed that the higher plasma levels of SNCA in humans and rodents models were associated with glucose metabolism and improved insulin response. Indeed, SNCA had a positive effect on glucose metabolism through the lyso-phosphatidic acid receptor (LPAR2). They have suggested that the SNCA can be associated with glucose uptake, reduced insulin resistance, and T2D [1]. According to the extracellular interaction between SNCA and LPAR2, it seems that SNCA may have an endocrine role, in addition to its intracellular activities. Since the role of SNCA has shown in glucose uptake in adipose and muscle cells, in present study we examined the effect of insulin resistance on the *SNCA* expression in the muscle C2C12 cell line and skeletal muscle tissues of diabetic mice with insulin resistance. The obtained results showed a significant negative correlation between *SNCA* expression and insulin resistance in both in vitro and in vivo studies.

Some studies have focused on the relationship between Parkinson’s disease and diabetes mellitus. It has shown that the SNCA aggregation as the main component of Lewy bodies increases the cell toxicity in dopaminergic neurons which can be the probable missing circle of diabetes progression in Parkinson’s disease. An in vitro study on dopaminergic N27 cell line has shown that the paraquat (PQ) associated cell toxicity was significantly reduced by glucose deprivation. On the other hand, the overexpression of wild type or A53T mutant α-synuclein and its interaction with PQ can stimulate glucose uptake through increasing levels of GLUT4 and sodium–glucose transporter 1 (SGLT1). Dopaminergic cell death was affected by PQ–SNCA interaction which is associated with glucose metabolism and adenosine monophosphate-activated protein kinase (AMPK) pathway [20]. Furthermore, advanced glycated end products (AGEs) in diabetic patients also elevate the glycated SNCA and its aggregation in dopaminergic neurons [21]. Another study has also shown that the SNCA–Rab8 interaction may increase the transfer of vesicles containing GLUT4 to the cell surface of dopaminergic cells [9].

## Conclusions

In the present study, we showed that the *SNCA* expression was decreased in insulin resistant C2C12 cells and also skeletal muscle tissues of diabetic mice with insulin resistance. These results support the studies that show the decreased levels of plasma SNCA in insulin resistant diabetic patients. According to the reduced *SNCA* expression in the muscle cell following insulin resistance, the *SNCA* overexpression may improve the insulin-resistant cell response to insulin. It can be concluded that the SNCA can be dependent to insulin and may have important role in glucose metabolism in insulin-dependent cells.

## Data Availability

The datasets used and/or analyzed during the current study are available from the corresponding author on reasonable request.

## Abbreviations

GLUT4: glucose transporter-4
T2D: type-2 diabetes
SNCA: alpha-synucleins
NIDDM: non-insulin dependent diabetes mellitus
IR: insulin-resistant
IS: insulin-sensitive
GM: growth medium
DM: differentiation medium
B2M: beta 2 microglobulin
LPAR2: lyso-phosphatidic acid receptor
PQ: paraquat
SGLT1: sodium–glucose transporter 1
AMPK: adenosine monophosphate-activated protein kinase
